# Subtilosin A production is influenced by surfactin levels in *Bacillus subtilis*

**DOI:** 10.1093/femsml/uqae029

**Published:** 2025-01-02

**Authors:** Caja Dinesen, Manca Vertot, Scott A Jarmusch, Carlos N Lozano-Andrade, Aaron J C Andersen, Ákos T Kovács

**Affiliations:** DTU Bioengineering, Technical University of Denmark, 2800 Kgs Lyngby, Denmark; Institute of Biology, Leiden University, 2333 BE Leiden, The Netherlands; DTU Bioengineering, Technical University of Denmark, 2800 Kgs Lyngby, Denmark; DTU Bioengineering, Technical University of Denmark, 2800 Kgs Lyngby, Denmark; DTU Bioengineering, Technical University of Denmark, 2800 Kgs Lyngby, Denmark; DTU Bioengineering, Technical University of Denmark, 2800 Kgs Lyngby, Denmark; DTU Bioengineering, Technical University of Denmark, 2800 Kgs Lyngby, Denmark; Institute of Biology, Leiden University, 2333 BE Leiden, The Netherlands

**Keywords:** *Bacillus subtilis*, secondary metabolite, surfactin, MALDI-MSI, subtilosin A, chemical ecology

## Abstract

Although not essential for their growth, the production of secondary metabolites increases the fitness of the producing microorganisms in their natural habitat by enhancing establishment, competition, and nutrient acquisition. The Gram-positive soil-dwelling bacterium, *Bacillus subtilis*, produces a variety of secondary metabolites. Here, we investigated the regulatory relationship between the non-ribosomal peptide surfactin and the sactipeptide bacteriocin subtilosin A. We discovered that *B. subtilis* mutants lacking surfactin production exhibited higher production of subtilosin A compared to their parental wild-type strain. Additionally, spatial visualization of *B. subtilis* production of metabolites demonstrated that surfactin secreted by a wild-type colony could suppress subtilosin A production in an adjacent mutant colony lacking surfactin production. Reporter assays were performed using mutants in specific transcriptional regulators, which confirmed the role of ResD as an activator of the subtilosin A encoding biosynthetic gene cluster (BGC), while the removal of Rok and AbrB repressors increased the expression of the BGC, which was further enhanced by additional deletion of surfactin, suggesting that a so-far-unidentified regulator might mediate the influence of surfactin on production of subtilosin A. Our study reveals a regulatory influence of one secondary metabolite on another, highlighting that the function of secondary metabolites could be more complex than its influence on other organisms and interactions among secondary metabolites could also contribute to their ecological significance.

## Introduction

Biosynthetic gene clusters (BGCs) that are involved in secondary metabolite (SM) production are prevalent across bacterial genera (Medema et al. 2011, Katz and Baltz 2016). While the production of SMs may not be essential in laboratory settings (Craney et al. 2013), they likely play a crucial role in the establishment of bacteria within natural niches (Hibbing et al. 2010, Giubergia et al. 2016). In the past, the role of SMs in nature has predominately been classified as microbial weapons, likely due to the industrial use of SMs to combat microbial infections (Demain and Fang 2000, Bode et al. 2002, O'Brien and Wright 2011). However, in recent years, this notion has been adjusted. While the antimicrobial properties of SMs are still acknowledged, more research is being directed toward understanding their ecological function rather than being a direct inhibitor of cellular processes (Yim et al. 2007, Ryan and Dow 2008, Straight and Kolter 2009, Sharrar et al. 2020). The soil-dwelling, plant growth-promoting bacterium, *Bacillus subtilis*, harbors a diverse array of BGCs, with surfactin and plipastatin being the most studied non-ribosomal lipopeptides (Beauregard et al. 2013, Kiesewalter et al. 2021, Schoenborn et al. 2021). Particularly, surfactin has a strong biosurfactant activity in addition to its antimicrobial properties (Pérez-Gil and Keough 1998, Sabaté and Audisio 2013, Zhao et al. 2017). Surfactin facilitates *B. subtilis* motility through swarming and sliding, thereby playing an important role in *B. subtilis* root colonization in soil (Grau et al. 2015, Gao et al. 2016, Jautzus et al. 2022). In addition to non-ribosomally synthesized peptides (NRPs), *B. subtilis* also produces ribosomally synthesized and post-translationally modified peptides (RiPPs). One of the *B. subtilis*-specific RiPPs, the bacteriocin subtilosin A, was first isolated in 1985 (Babasaki et al. 1985) and it displays antibacterial activity toward both Gram-positive and Gram-negative bacteria (Babasaki et al. 1985, Zheng et al. 1999, Shelburne et al. 2007). Other functions of subtilosin A have been reported such as suppression of biofilm formation in *Listeria monocytogenes, Gardnerella vaginalis*, and *Escherichia coli* (Algburi et al. 2017). Furthermore, Schoenborn et al. found delayed sporulation in a mutant lacking subtilosin A compared to its parental wild-type strain (Schoenborn et al. 2021).

Whereas surfactin production has been extensively studied across a plethora of *B. subtilis* isolates, research on the production of subtilosin A has predominantly concentrated on the domesticated *B. subtilis* 168 strain or its derivative JH642 (Babasaki et al. 1985, Zheng et al. 1999, Stein et al. 2004). Domesticated *B. subtilis* strains lack surfactin production due to mutation in the *sfp* gene (Kearns et al. 2004). Importantly, natural isolates of *B. subtilis* encode the intact BGC for subtilosin A production (BGC^Sbo^) (Kiesewalter et al. 2021) and the presence of this BGC is fully conserved among all isolates of *B. subtilis* (Steinke et al. 2021); nevertheless, the production of subtilosin A has not been reported in undomesticated strains.

BGC^Sbo^, the *sbo–alb* operon, encodes the proteins SboA, SboX, and AlbA–AlbG involved in post-translational modifications, processing, and export of the peptide, respectively (Zheng et al. 1999). BGC^Sbo^ is regulated by several transcription factors, including Rok, AbrB, and ResD, in addition to the sigma factor SigA. Rok and AbrB repress, while ResD activates the expression of BGC^Sbo^ (Strauch et al. 2007). Production of subtilosin A is linked to later growth stages, characterized by nutrient starvation and oxygen limitation (Stein 2020, Schoenborn et al. 2021). Nakano and colleagues demonstrated that the two-component response regulator, ResDE, is essential for activating the subtilosin A BGC in response to oxygen limitation (Nakano et al. 2000).

Several starvation or stationary phase-specific genes are repressed during exponential growth by AbrB, which directly binds to the respective promotors of those genes, as demonstrated for the *sboA* gene. AbrB-mediated repression is alleviated by Spo0A during starvation (Hahn et al. 1995). Additionally, AbrB also represses the transcription of *rok* (Hoa et al. 2002, Albano et al. 2005). Similarly, Rok binds directly to the promoter of *sboA* and represses its expression (Albano et al. 2005). While no specific signal or environmental condition has been correlated with the activity of Rok, it is noteworthy that sRok, an interaction partner of Rok, exhibits altered binding affinity during salt stress (Erkelens et al. 2022). sRok and DnaA, another interaction partner of Rok, affect the binding affinity of Rok, which may affect Rok’s regulatory role (Erkelens et al. 2024). Moreover, Rok regulates several genes (Albano et al. 2005, Smits and Grossman 2010), including *sboA*, as well as the biofilm gene *bslA* (*yuaB*) in *B. subtilis* (Kovács and Kuipers 2011). While these studies provide detailed molecular insights into the transcriptional regulation of BGC^Sbo^, the regulation and production of subtilosin A have only been explored in *B. subtilis* 168 and its derivatives that lack surfactin production, excluding any insights into potential co-dependencies or conflicting expression related to subtilosin A and surfactin production.

In this study, we demonstrate that while the two SMs, surfactin and subtilosin A, are not produced simultaneously, the presence of surfactin regulates the production of subtilosin A in *B. subtilis*. Additionally, we investigate the regulatory mechanism by which surfactin suppresses the expression of BGC^Sbo^ using knockout mutants in gene encoding transcriptional regulators. Employing GFP reporter assays, analytical chemistry, and spatial detection of SMs, we demonstrate that extracellular surfactin inhibits the production of subtilosin A in mutants that otherwise lack surfactin production.

## Materials and methods

### Bacterial strains and culture media

All strains used in this study, including genomic DNA (gDNA) donors, are listed in Table 1. Overnight starter cultures were grown in lysogeny broth (LB; Carl Roth, Germany; 10 g l^−1^ tryptone, 5 g l^−1^ yeast extract, and 5 g l^−1^ NaCl) medium. If not stated otherwise, experiments were performed in potato dextrose broth (PDB; BD, USA; potato infusion at 4 g l^−1^, glucose at 20 g l^−1^), supplemented with 1.5% agar when required.

**Table 1.** Detailed information about strains used in this study.

Strain	Description	Reference
168	*amyE*::P*_sboA_– gfp* (Cm^R^)	Mhatre et al. (2016)
DK1042	NCIB 3610 *coml*^Q12^	Konkol et al. (2013)
DS1122	3610 Δ*srfAC* (Mls^R^)	Chen et al. (2009)
DS4114	3610 Δ*ppsC* (Tet^R^)	(Müller et al. 2014)
DS3337	3610 Δ*sfp* (Mls^R^)	Patrick and Kearns (2009)
P8_B1	WT	Kiesewalter et al. (2021)
P8_B1	Δ*srfAC* (Mls^R^)	Kiesewalter et al. (2021)
P8_B1	Δ*ppsC* (Tet^R^)	Kiesewalter et al. (2021)
P8_B1	Δ*sfp* (Mls^R^)	Kiesewalter et al. (2021)
DTUB366	DK1042 *amyE*::P*_sboA_*–*gfp* (Chl^R^)	This study
DTUB367	DK1042 *amyE*::P*_sboA_*–*gfp* (Chl^R^); Δ*srfAC* (Mls^R^)	
DTUB368	DK1042 *amyE*::P*_sboA_*–*gfp* (Chl^R^); Δ*ppsC* (Tet^R^)	
DTUB369	DK1042 *amyE*::P*_sboA_*–*gfp* (Chl^R^); Δ*sfp* (Mls^R^)	
DTUB370	DK1042 *amyE*::P*_sboA_*–*gfp* (Chl^R^); Δ*rok* (Km^R^)	
DTUB371	DK1042 *amyE*::P*_sboA_*–*gfp* (Chl^R^); Δ*resD* (Km^R^)	
DTUB372	DK1042 *amyE*::P*_sboA_*–*gfp* (Chl^R^); Δ*abrB* (Km^R^)	
DTUB373	DK1042 *amyE*::P*_sboA_*–*gfp* (Chl^R^); Δ*rok* (Km^R^), Δ*srfAC* (Mls^R^)	
DTUB374	DK1042 *amyE*::P*_sboA_*–*gfp* (Chl^R^); Δ*resD* (Km^R^), Δ*srfAC* (Mls^R^)	
DTUB375	DK1042 *amyE*::P*_sboA_*–*gfp* (Chl^R^); Δ*abrB* (Km^R^), Δ*srfAC* (Mls^R^)	

### Generation of mutant *B. subtilis* strains

DK1042 P*_sboA_–gfp* was obtained with gDNA from the gDNA donor 168 *amyE::*P*_sboA_–gfp*. Mutant strains in DK1042 P*_sboA_–gfp* were obtained by natural competence (Anagnostopoulos and Spizizen 1961), by transforming gDNA and selecting for antibiotic (AB) resistance on AB containing LB agar medium. gDNA was extracted from the donors using the EURx Bacterial & Yeast Genomic DNA Purification Kit (EURx, Gdansk, Poland), following the manufacturer’s instructions. To verify transformation and lack of SM production, overnight grown cultures were directly extracted with acetonitrile using a 1:1 acetonitrile:culture dilution, then the solution was centrifuged and supernatant transferred to high-performance liquid chromatography (HPLC) vials and analyzed by ultrahigh-performance liquid chromatography coupled to high-resolution mass spectrometry (UHPLC-HRMS).

### Expression assay in *B. subtilis* BGC mutants

The effect of SM production on the expression BGC^Sbo^ was evaluated in plate reader assays. Fluorescence and optical density were detected in cultures grown in 96-well microtiter plates with 200 µl PDB, including the reporter strains with a final optical density of 0.01 at 600 nm (OD_600_). To test the influence of surfactin on the expression of *sboA*, a similar setup was used, except that the P*_sboA_*–*gfp* Δ*srfAC* strain was supplemented with surfactin at final concentrations of 50, 100, 200 and 400 µg ml^−1^. PDB medium without surfactin served as a control. Cultivation was performed in a Synergy XHT Multimode Reader (BioTek Instruments, Winooski, VT, US) at 30°C with orbital continuous shaking (3 mm), monitoring the OD_600_ as well as GFP (Ex: 482/20; Em: 528/20; Gain: 60) fluorescence every 5 min.

### Detection of subtilosin A and surfactin in neighboring colonies of wild-type and Δ*srfAC* strains

Complementation of surfactin production by the wild-type colony toward the neighboring Δ*srfAC* mutant was tested on PDA medium. A volume of 2 µl of overnight grown bacterial cultures were inoculated on PDA medium using a 2.5 cm distance between the inoculation points of the two strains. The plates were incubated at 37ºC for 3 d. To assess the level of surfactin and subtilosin A, four plugs were transferred from the plates distributed from the distal region of the Δ*srfAC* colony to the distal edge of the wild-type colony. A volume of 1.5 ml of isopropanyl ethyl acetate (1.3 v/v) with 1% formic acid was added to each plug and sonicated for 60 min before centrifugation (3 min, 13 400 rpm). The supernatant was extracted and transferred under N_2_ with no heat before resuspension in 250 µl methanol and centrifugation (3 min, 13 400 rpm). Supernatant was transferred to HPLC vials and tested by UHPLC-HRMS.

UHPLC-HRMS was performed on an Agilent Infinity 1290 UHPLC system with a diode array detector. UV–visible spectra were recorded from 190 to 640 nm. Liquid chromatography of 1 µl extract (or standard solution) was performed using an Agilent Poroshell 120 phenyl-hexyl column (2.1 × 150 mm, 1.9 µm) at 40°C with acetonitrile (ACN) and H_2_O, both containing 20 mM formic acid, as mobile phases. Initially, a gradient of 10% ACN/H_2_O to 100% acetonitrile over 10 min was employed, followed by isocratic elution of 100% ACN for 2 min. The gradient was returned to 10% ACN/H_2_O in 0.1 min, and, finally, to an isocratic condition of 10% ACN/H_2_O for 2.9 min, at a flow rate of 0.35 ml min^−1^. HRMS spectra were acquired in positive ionization mode on an Agilent 6545 quadrupole time-of-flight mass spectrometry (QTOF MS) equipped with an Agilent Dual Jet Stream electrospray ion source with a drying gas temperature of 250°C, drying gas flow of 8 l min^−1^, sheath gas temperature of 300°C, and sheath gas flow of 12 l min^−1^. Capillary voltage was set to 4000 V and nozzle voltage to 500 V. MS data analysis and processing were performed using Agilent MassHunter Qualitative Analysis B.07.00.

### Mass spectrometry imaging of pairwise interactions between Δ*srfAC* and wild-type colonies

Samples were prepared as described above for quantification of SMs from PDA grown colonies. The interaction zone of the two colonies was excised from agar plates and adhered to MALDI IntelliSlides (Bruker, Billerica, MA, USA) using a 2-Way Glue Pen (Kuretake Co., Ltd, Nara-Shi, Japan). Slides were covered by spraying 1.5 ml of 2,5-dihydrobenzoic acid (40 mg ml^−1^ in MeOH/H_2_O (80:20, v/v, 0.1% trifluoroacetic acid)) and dried prior to MSI acquisition. Matrix-assisted laser desorption/ionization mass spectrometry imaging (MALDI-MSI) data were acquired using a timsTOF fleX (Bruker Daltonik GmbH) mass spectrometer operating in a positive mode with a rater width of 30 µm and an *m/z* range of 500–4000. Calibration was performed by using red phosphorus. The settings in the timsControl were as follows: laser: imaging 30 µm, power boost 3.0%, scan range 26 µm in the XY interval, and laser power 70%; tune: funnel 1 RF 300 Vpp, funnel 2 RF 300 Vpp, multipole RF 300 Vpp, isCID 0 eV, deflection delta 70 V, MALDI plate offset 100 V, quadrupole ion energy 5 eV, quadrupole loss mass 100 *m/z*, collision energy 10 eV, focus pre-TOF transfer time 75 µs, and pre-pulse storage 8 µs. Data were root mean square normalized and visualized in SCiLS software (Bruker Daltonik GmbH, Bremen, Germany).

### Statistics

Data were analyzed and graphically represented using R 4.3.2 and the package ggplot2 (Wickham 2016). Student’s *t*-test was used to test for statistical differences in experiments between two groups. Statistical significance (α) was set at 0.05. For multiple comparisons (more than two treatments), ANOVA and Tukey’s honestly significant difference (HSD) were performed. In all the cases, normality and equal variance were assessed using the Shapiro–Wilk and Levene tests, respectively.

## Results

### Production of subtilosin A is increased in mutant strains lacking surfactin

**Figure 1.**
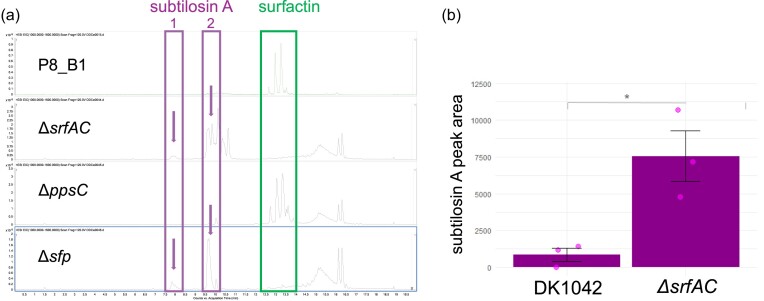
(a) LC–MS chromatogram (EIC: *m/z* 1000–1600) for *B. subtilis* P8_B1 and its derivative mutants Δ*srfAC* (lacking surfactin), Δ*ppsC* (lacking plipastatin), and Δ*sfp* (lacking all NRPs). Subtilosin A’s peak (1134.1963 [M+3H]^3+^) is highlighted in the purple boxes. The box labeled with 1 highlights the hydrolyzed form of subtilosin A, while box 2 refers to the cyclized subtilosin A product. The isomers of surfactin are highlighted with the green box. (b) The production of subtilosin A in DK1042 and Δ*srfAC* estimated by peak area from EIC data, and statistical difference was tested using students *t*-test (*P* = 0.0193, *t*-test, *n* = 3).

We previously investigated SM production in 12 natural isolates of *B. subtilis* and tested mutants unable to produce specific NRPs (Kiesewalter et al. 2021). Analysis of SM production in one of these isolates, P8_B1, and its derivative NRP-related BGC mutants using liquid chromatography–mass spectrometry (LC–MS) revealed a varying presence of subtilosin A between P8_B1 and mutants (Fig. 1a). The chemical extractions originating from the mutant derivatives lacking surfactin production (Δ*srfAC* and Δ*sfp*) displayed an additional LC–MS peak corresponding to an *m/z* of 1134.1963 ([M+3H]^3+^) that was identified as subtilosin A (National Center for Biotechnology Information 2024). The same peak is observed in the LC–MS profiles of other isolates that correspond to mutants lacking surfactin production (Kiesewalter et al. 2021). We previously validated that the LC–MS peaks correspond to surfactin (Kiesewalter et al. 2021), which is highlighted with the green box in Fig. 1a. Indeed, LC–MS peaks corresponding to surfactin are absent in the Δ*srfAC* and Δ*sfp* strains. To confirm this observation in the most frequently used undomesticated *B. subtilis* strain (DK1042 the naturally competent derivative of NCIB3610), samples were extracted from strains DK1042 and Δ*srfAC* to quantify the level of subtilosin A using the peak area. This approach showed an 8.7-fold increase between the peak area in the mutant strain compared to the wild-type DK1042 (*P* = 0.0193, *t*-test) (Fig. 1b).

### Surfactin attenuates the expression of BGC^Sbo^

**Figure 2.**
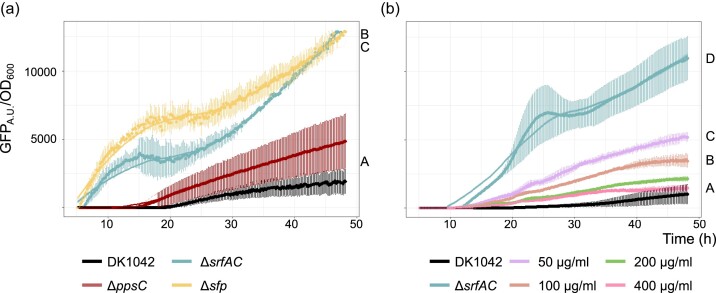
(a) Expression of BGC^Sbo^ in wild-type and derived BGC mutants compared using corresponding strains carrying P*_sboA_*–*gfp* reporter fusion. The fluorescence was normalized by growth (optical density at 600 nm, OD_600_). (b) Expression of BGC^Sbo^ in the Δ*srfAC* strain carrying P*_sboA_*–*gfp* reporter fusion supplemented with varying concentrations of surfactin (50–400 µg ml^–1^). Normalized GFP expression between different strains and treatments was compared using the area under the curve (AUC) using one-way ANOVA and Tukey’s honest test. Letters present significant differences between strains (Table S1).

To determine whether the lack of subtilosin A in LC–MS samples from surfactin producers was due to differentiated production or degradation of subtilosin A, we tested the expression of BGC^Sbo^ in the wild-type and mutant derivatives using the GFP signal normalized by OD_600nm_ as proxy. For this, the promoter region of the *sboA* gene of BGC^Sbo^ was inserted before the promoter-less *gfp* gene and the construct was introduced into the *amyE* locus of the wild-type and mutant strains (see the “Materials and methods” section). Green fluorescence was followed in plate reader assays (see the “Materials and methods” section). Here, the expression of BGC^Sbo^ was increased in both the Δ*srfAC* and the Δ*sfp* strains compared to the wild type [*P* ≤ 0.0001, *P* ≤ 0.0001, one-way analysis of variance (ANOVA) and Tukey’s honestly significant difference (HSD)] (Fig. 2a). To evaluate whether the influence of the lack of surfactin production can be extracellularly complemented, commercially available purified surfactin was supplemented to the Δ*srfAC* strain in varying concentrations, showing a reduction in the expression of BGC^Sbo^ with increasing concentration of surfactin (Fig. 2b). Externally added surfactin at a concentration of 400 µg ml^−1^ almost reduced the BGC^Sbo^ expression level in the Δ*srfAC* strain to the levels observed in the wild type (*P* = 0.8196, ANOVA and Tukey’s HSD).

### Complementation of diminished surfactin production in the Δ*srfAC* mutant colony by a neighboring wild-type colony

**Figure 3.**
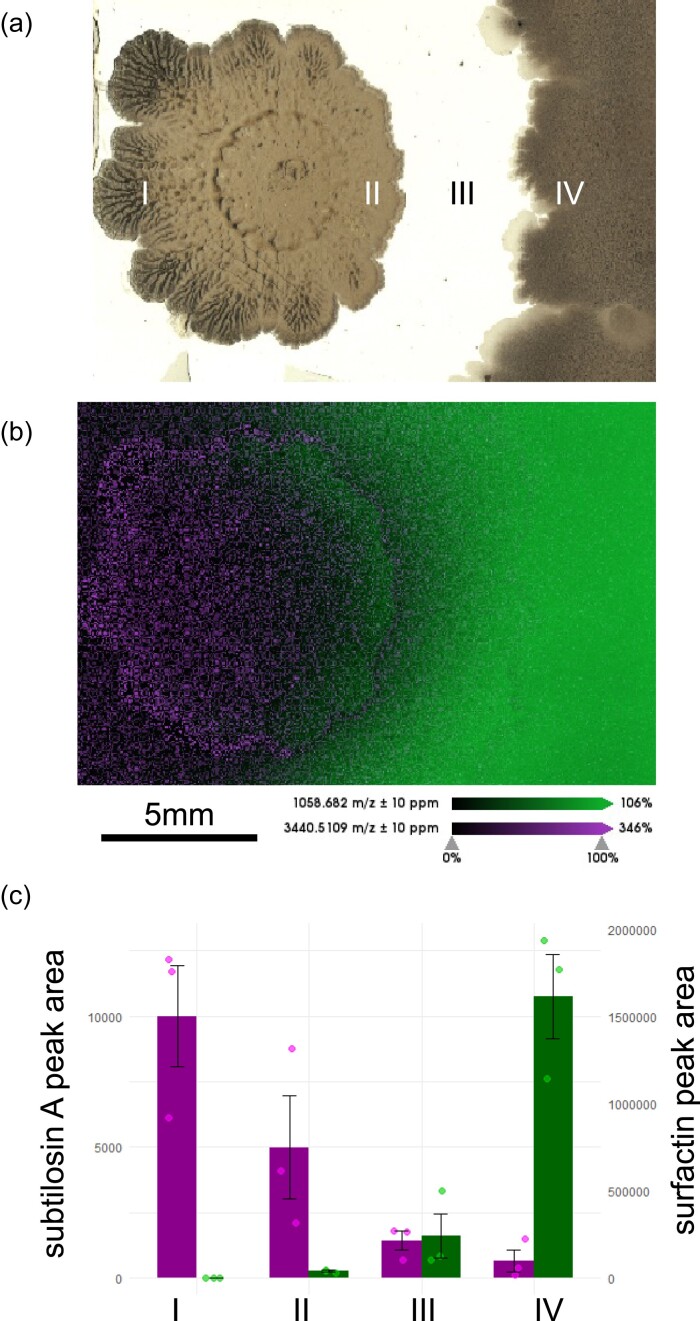
Spatial mapping of subtilosin A and surfactin distribution in neighboring colonies. (a) Light image of Δ*srfAC* (left) and wild-type (right) colonies, including the approximate positions of samples taken for LC–MS analysis (I–IV) on a replicate. Scale bar indicates 5 mm. (b) MALDI-MSI-based localization of subtilosin A (magenta) and surfactin (green) in the neighboring colonies of Δ*srfAC* and wild-type strains. (c) Relative amount of subtilosin A (magenta) and surfactin (green) estimated by peak area from the LC–MS EIC data in the samples taken at different positions depicted in panel (a).

As external complementation with surfactin can reduce the BGC^Sbo^ expression in the Δ*srfAC* strain similar to the levels seen in the wild type, we investigated whether surfactin production by a wild-type colony could downregulate the expression of BGC^Sbo^ in a neighboring Δ*srfAC* colony. Wild-type and Δ*srfAC* strains were spotted next to each other on potato dextrose agar (PDA) medium and sampled for visual detection of SMs. Spatial mapping of metabolites allowed visualization of surfactin production and secretion into the agar by the wild-type strain reaching the proximal edge of the Δ*srfAC* colony. Subtilosin A was detected in a reverse distribution, with high abundance on the distal part of the Δ*srfAC* colony (zone I) with a gradual decrease toward the wild-type neighboring edge of the colony (zone II) (Fig. 3a and b). Samples were harvested in a line crossing the middle of both colonies (I–IV) and subjected to semi-quantitative LC–MS analysis that verified the diffusion of surfactin from the wild-type strain in its environment, in addition to gradually decreasing subtilosin A levels in the Δ*srfAC* colony at increasing surfactin concentrations (Fig. 3c). Additionally, analysis of the spatial metabolite distribution also revealed that the production of the sporulation killing factor (SKF) was absent in the Δ*srfAC* colony, while it was abundant in the wild-type strain (Fig. S1).

### Influence of lack of surfactin production on regulation of BGC^Sbo^ by known global regulators

**Figure 4.**
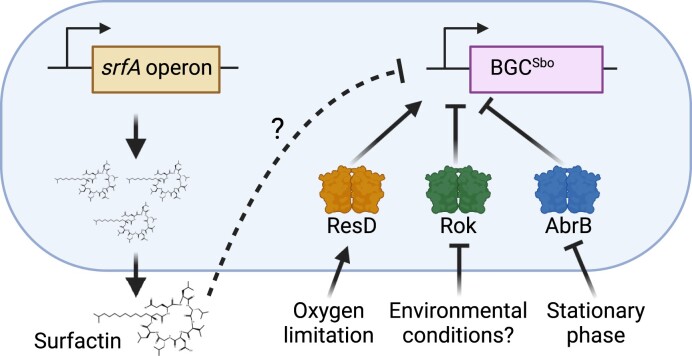
Schematic representation of BGC^Sbo^ expression in *B. subtilis*. BGC^Sbo^ expression is transcriptionally regulated by three known regulators: ResD, Rok, and AbrB, which respond to various signals. The figure also depicts the influence of surfactin on BGC^Sbo^ expression. Arrows indicate production and activation, while T lines denote repression. Dashed line refers to either direct or indirect influence.

**Figure 5.**
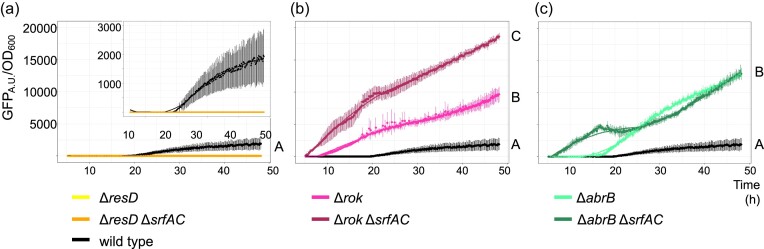
Expression of BGC^Sbo^ in wild-type (black line) and derived regulator mutants compared using corresponding strains carrying P*_sboA_*–*gfp* reporter fusion. The fluorescence was normalized by growth (OD_600_). Expression was assayed in Δ*r esD* (a), Δ*rok* (b), and Δ*abrB* (c) single mutants (light line colors) or in combination with Δ*srfAC* (dark line colors). Normalized GFP expression between different strains and treatments was compared using the AUC by employing one-way ANOVA and Tukey’s honest test. Letters represent significant differences between strains (Table S2).

To evaluate whether surfactin downregulates BGC^Sbo^ expression through one of the known global regulators of BGC^Sbo^, *resD, rok*, and *abrB* genes (Fig. 4) were disrupted in the wild type and Δ*srfAC* carrying P*_sboA_*–*gfp*. Deletion of *resD* prevented the expression of *sboA* (Fig. 5a), whereas introduction of Δ*rok* and Δ*abrB* increased expression of BGC^Sbo^ (*P* ≤ 0.0001 and *P* ≤ 0.0001, ANOVA and Tukey ’sHSD) (Fig. 5b and c). The combination of Δ*srfAC* with Δ*resD* did not influence the already diminished expression of BGC^Sbo^ (Fig. 5a). In contrast, deletion of the BGC for surfactin production in Δ*rok* further increased the expression level of BGC^Sbo^ (*P* ≤ 0.0001, ANOVA and Tukey’s HSD) (Fig. 5b). While this increase was maintained throughout the experiment in the absence of the *rok* gene, BGC^Sbo^ expression in the Δ*abrB* Δ*srfAC* mutant was only enhanced in the first 20 h, whereas afterward it was comparable to that in the single Δ*abrB* strain with no statistical difference (*P* = 0.5463, ANOVA and Tukey’s HSD) (Fig. 5c).

## Discussion

SMs have been extensively investigated and harnessed, playing a pivotal role in combating microbial infections and improving human health (Hibbing et al. 2010, Giubergia et al. 2016), with a growing interest in the application of SMs beyond medicine (Bode et al. 2002, O'Brien and Wright 2011). In particular, understanding the ecological functions of SMs can enhance the utilization of SM-producing bacteria in agricultural applications, where production of SM is important for the efficiency of biocontrol bacteria. Identification of the underlying regulatory mechanisms influencing SM production may facilitate elucidating their role in nature.

Here, we dissected the influence of the lipopeptide surfactin on expression and production of the bacteriocin subtilosin A in *B. subtilis*. Surfactin decreased the production of subtilosin A in *B. subtilis*, while strains lacking surfactin production had an increased level of subtilosin A. The lack of surfactin production and therefore enhanced subtilosin A level could be reverted by pure surfactin or inoculating a neighboring wild-type colony next to the Δ*srfAC* mutant strain. Testing the expression of BGC^Sbo^ demonstrated a transcriptionally regulatory mechanism behind surfactin-mediated repression of subtilosin A production. Previous studies have demonstrated both overlapping and dissimilar production of SMs in *B. subtilis* (Yannarell et al. 2023). For example, Yannarell et al. (2023) reported a little overlap of cells expressing both BGCs for surfactin and subtilosin A production in biofilm colonies. Spatial detection of key SMs in *B. subtilis* biofilm colonies has been previously reported using MALDI-MSI (Si et al. 2016). Although not specifically reported, the MALDI-MSI images suggest increased subtilosin A production in the Δ*srfAA* mutant colony, confirming our results. Similarly, a reduced SKF level was noticeable in the Δ*srfAA* mutant used by Si and colleagues (Si et al. 2016), which again confirms our data.

The lack of simultaneous production of surfactin and subtilosin A might suggest that their roles in *B. subtilis* are distinctive and these SMs might contribute to different developmental stages or specific environmental conditions. Or rather that in the absence of surfactin, subtilosin A antibacterial properties are replacing those of surfactins. While experimental validation is required to demonstrate such possibility, various roles of RiPPs have previously been reported, such as growth inhibition, nutrient competition, and quorum sensing (Li and Rebuffat 2020). Notably, surfactin plays a pivotal role in the early stages of root colonization in soil and during initiation of biofilm formation (Grau et al. 2015, Gao et al. 2016, Jautzus et al. 2022), congruent with the early exponential phase expression of the *srfA* operon, around 7 h after inoculation (Maan et al. 2021). In contrast, production of subtilosin A is correlated with the end of the exponential phase/starting stationary phase (Stein 2020). The quantities in which *B. subtilis* produce these SMs are also different. The level of surfactin has been quantified in different *B. subtilis* strains, ranging from 1.25 to 6.45 g l^−1^ (Yeh et al. 2005, Abdel-Mawgoud et al. 2008, Amani et al. 2013, Zhen et al. 2023), while subtilosin A concentration in different strains and conditions has been reported to be between 0.5 and 7.8 mg l^−1^ (Babasaki et al. 1985, Stein 2020). The production of surfactin and subtilosin A was not measured quantitatively in our study; however, the LC–MS data suggest that surfactin was produced in higher quantities than subtilosin A. The difference in production quantity might be related to their role in the environment, since the function of surfactin as a bio-surfactant may require higher quantities compared to the primarily antibiotic role of subtilosin A.

The gene cluster encoding subtilosin A synthesis is known to be regulated by the global regulators ResD, Rok, and ArbB (Strauch et al. 2007). Our analysis with *sboA* promoter coupled *gfp* reporter strains confirmed current knowledge on the role of ResD, Rok, and AbrB in the transcriptional regulation of BGC^Sbo^ (Strauch et al. 2007). Disruption of surfactin production further increased the expression of BGC^Sbo^ on a Δ*rok* background, suggesting that Rok is not involved in perceiving the presence of surfactin. Since ResD works as an activator of BGC^Sbo^ expression, deletion of both *resD* and *srfAC* does not permit the demonstration of whether surfactin influences ResD. AbrB functions as a repressor of BGC^Sbo^ transcription, with its repression being relieved during starvation. While disruption of surfactin production in a Δ*abrB* background hastened the expression of BGC^Sbo^in the first 20 h compared to the single deletion of *abrB*, the expression levels of BGC^Sbo^ were comparable in the two strains from 20 h onward. The enhanced expression of BGC^Sbo^ observed in the earlier phase of the population growth in a Δ*abrB* background, when the expression of surfactin-related BGC is prominent, suggests that surfactin is not regulating subtilosin A production through AbrB. Interestingly, while deletion of either *srfAC, sfp*, or *rok* increases BGC^Sbo^ expression from the first few hours of the population growth, disruption of *abrB* only influences BGC^Sbo^ expression 20 h after inoculation of the culture. These experiments suggest that an additional regulatory system might be involved in perceiving the presence of surfactin in *B. subtilis*. It is possible that surfactin influence membrane fluidity that results in downregulation of BGC^Sbo^expression. This is also suggested by slightly increased BGC^Sbo^ expression in the strain lacking plipastatin, the other lipopeptide produced by *B. subtilis* in addition to surfactin. Indeed, surfactin influences gene expression related to biofilm development of *B. subtilis* (López et al. 2009, Thérien et al. 2020, Stannius et al. 2024) and influences membranous structures in other microorganisms (Richter et al. 2024). Examination of the wild-type and Δ*srfAC B. subtilis* transcriptome could potentially reveal which genes and regulatory pathways are primarily influenced by surfactin. This could additionally reveal whether the transcription of BGCs other than BGC^Sbo^ is differentially regulated in the absence of surfactin, in accordance with the decreased SKF level detected in the Δ*srfAC* mutant colony.

Identifying possible correlations and differences in the production of SMs in *B. subtilis*, such as that described here between subtilosin A and surfactin, could further increase our understanding of the ecological roles of SMs.

## Supplementary Material

uqae029_Supplemental_File

## Data Availability

All raw data used to generate figures in the study are available from the corresponding author upon request.
