# Supplementary material for: Subtilosin A production is influenced by surfactin levels in Bacillus subtilis
Source: Microlife. 2025 Jan 2;6:uqae029. doi: 10.1093/femsml/uqae029 (PMC11756287; doi:10.1093/femsml/uqae029)
Supplement: uqae029_Supplemental_File [file uqae029_supplemental_file.pdf]

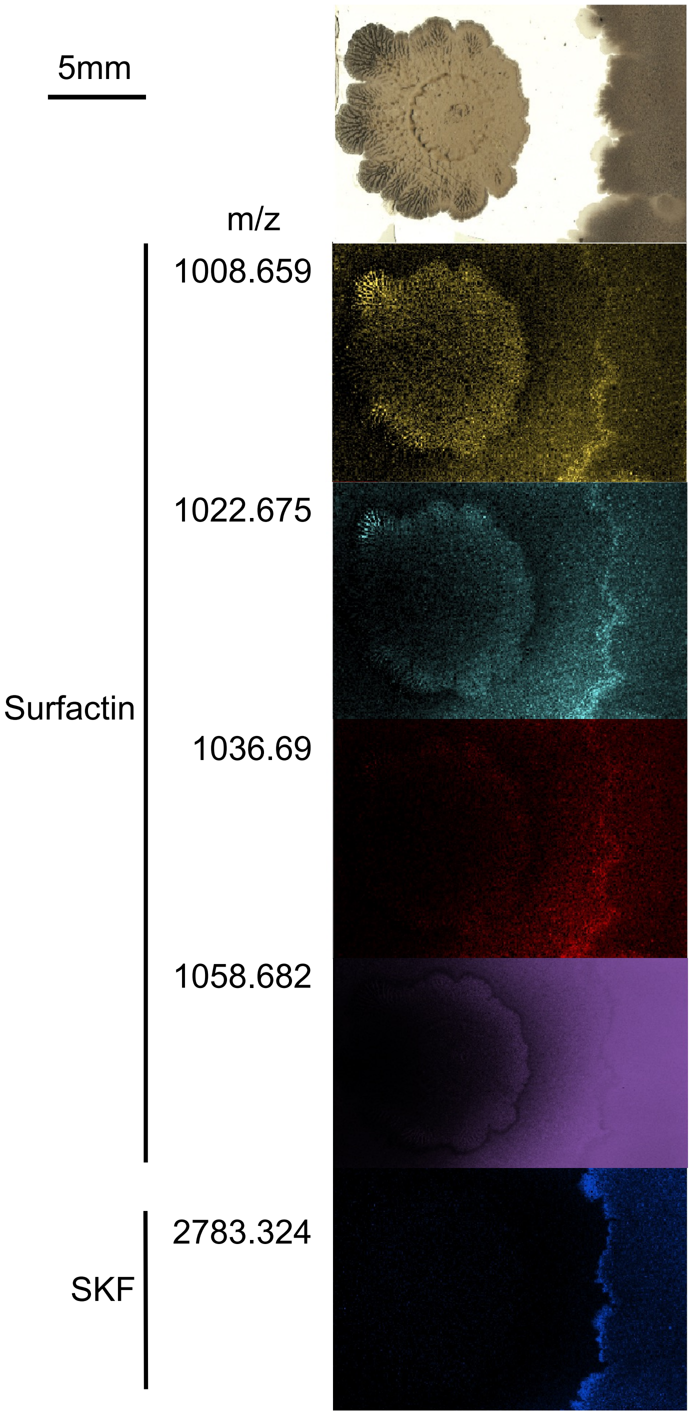

Fig. S1 Spatial mapping of surfactin isomer and SKF distribution in neighboring colonies. Top row includes the light image of Δ*srfAC* (left) and wild type (right) colonies. Scalebar indicates 5 mm. MALDI mass spectrometry imaging-based localization of surfactin isomers and SKF in the neighboring colonies of Δ*srfAC* and wild-type strains.

Table S1 Statistics on data used in Fig 2

Fig 2A			Fig 2B	
WT – *srfAC* ****	p <0.0001		WT – *srfAC* ****	p <0.0001
WT – *ppsC* ns	p = 0.115		WT – 50 ***	p = 0.0003
WT – *sfp* ****	p <0.0001		WT – 100 *	p = 0.0182
srfAC – *sfp* *	p = 0.0492		WT – 200 ns	p = 0.4597
			WT – 400 ns	p = 0.8196

Table S2 Statistics on data used in Fig 4

Fig 4A	
WT – *resD*	ns
WT – *resD*, *srfAC*	ns
resD – *resD*, *srfAC*	ns
Fig 4B	
WT – *rok* ****	p <0.0001
WT – *rok*, *srfAC* ****	p <0.0001
rok – *rok*, *srfAC* ****	p <0.0001
Fig 4C	
WT – *abrB* ****	p <0.0001
WT–*abrB*, *srfAC* ****	p <0.0001
abrB – *abrB*, *srfAC* ns	p = 0.5463
